# Correcting Flank Skin Laxity and Dog Ear Plus Aggressive Liposuction: A Technique for Classic Abdominoplasty in Middle-Eastern Obese Women

**Published:** 2018-01

**Authors:** Seyed Nejat Hosseini, Ali Ammari, Seyed Mehdi Mousavizadeh

**Affiliations:** 1 Department of Surgery, Ayatollah Mousavi Hospital, Zanjan University of Medical Sciences, Zanjan, Iran;; 2 Department of Plastic and Reconstructive Surgery, 15^th^ Khordad Hospital, Shahid Beheshti University of Medical Sciences, Tehran, Iran

**Keywords:** Liposuction, Flank skin laxity, Dog ear, Middle east, Women, Obesity

## Abstract

**BACKGROUND:**

Nowadays obesity is a common problem as it leads to abdominal deformation and people’s dissatisfaction of their own body. This study has explored using a new surgical technique based on a different incision to reform the flank skin laxity and dog ear plus aggressive liposuction on women with abdominal deformities.

**METHODS:**

From May 2014 to February 2016**, **25 women were chosen for this study. All women had a body mass index more than 28 kg/m^2^, flank folding, bulging and excess fat, abdominal and flank skin sagging and laxity. An important point of the new technique was that the paramedian perforator was preserved.

**RESULTS:**

All women were between 33 and 62 years old (mean age of 47±7.2 years old). The average amount of liposuction aspirate was 2,350 mL (1700-3200 mL), and the size of average excised skin ellipse was 23.62×16.08 cm (from 19×15 to 27×18 cm). Dog ear, skin laxity, bulging and fat deposit correction were assessed and scored in two and four months after the surgery.

**CONCLUSION:**

Aggressive abdominal and flank liposuction can be safely done when paramedian perforator is preserved. This has a good cosmetic result in the abdomen and flank and prevents bulging in the incision end and flank. Using this abdominoplasty technique is recommended on patients with high body mass indexes.

## INTRODUCTION

Nowadays, obesity is a common problem as it leads to abdominal deformation and people’s dissatisfaction of their own body. Abdominoplasty was first described in 1899 to treat umbilical hernia. It is not a cosmetic surgery only, because it secures integrity and firmness of the abdominal wall too. Its main goal is to correct the abdominal wall shape with minimum scars and preserve the natural umbilicus form.^1^^-^^4^

In 1924, the lower abdomen incision approach was adopted. This surgery is currently done with various techniques and incisions which still do not completely meet today’s patients’ demands. Patients who undergo this surgery complain about the long surgical incision, flanks and back scars, bulging in incision end (dog ears), abdominal sagging and laxity, and lack of lumbar curvature.^2^^,^^3^^,^^5^ Another problem of the classic abdominoplasty is the flat appearance, lacking the subtle anatomical features of a three-dimensional abdomen.^6^

**Fig. 1:**
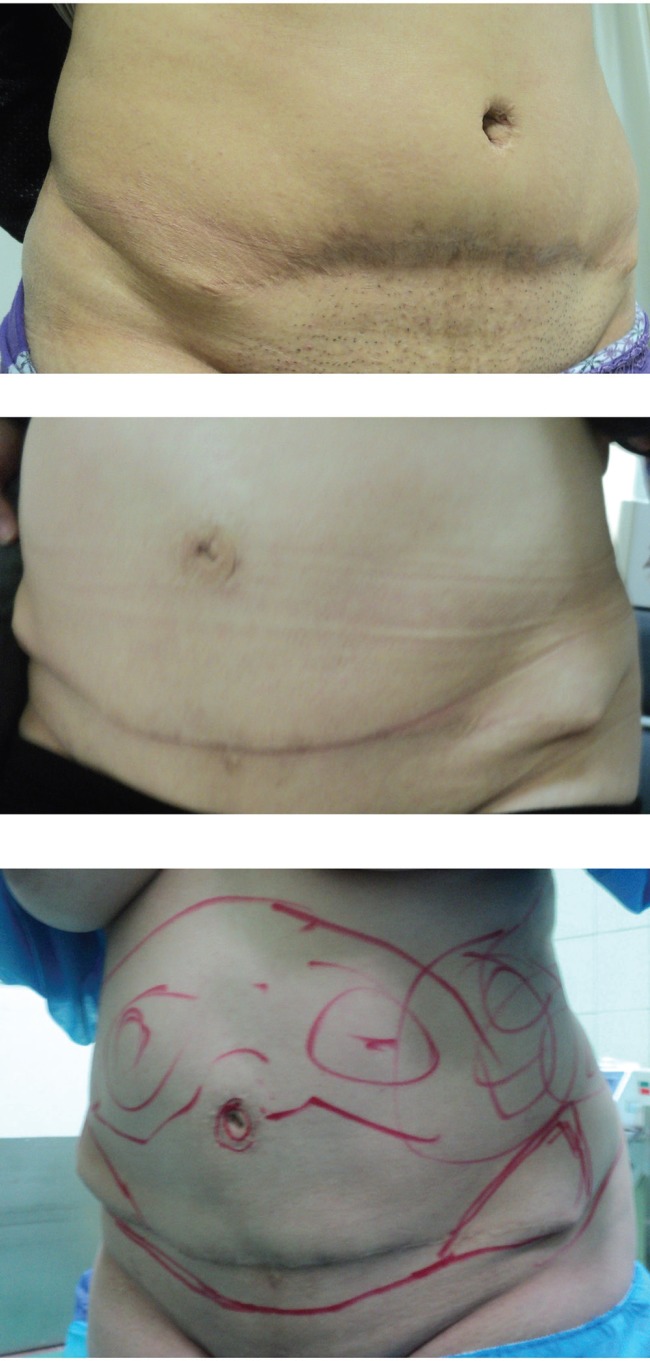
These patients had done classic abdominoplasty previously by other surgeons and their complications are obvious in the pictures which include: dog ear, fat deposit, unpleasant flank, and panhandle-shaped incision end bilaterally.

Although abdominoplasty is done with different methods, patients would still have post-surgical complaints, especially in obese patients (Figure 1). In spite of extensive investigations that are aimed to improve abdominoplasty results and correct surgical procedures (such as the reversed Y and W techniques), there are still post-surgical problems that bother the patients.^2^^,^^3^^,^^7^ These problems, which are the cause of complaints and increased post-abdominoplasty revisions, are mostly observed in Middle Eastern patients, who are mostly obese and have an average stature (high body mass index: BMI) (Figure 1). 

This study has explored using a new surgical technique based on a special incision to reform the flank skin laxity and dog ear plus aggressive liposuction on women with abdominal deformities. Then it has assessed its results of flank skin laxity, dog ear, proper flank curvature, surgical scars along incision lines, and patients’ satisfaction. 

## MATERIALS AND METHODS

This study was done on women who visited our center from May 2014 to February 2016. The inclusion criteria were (i) being a woman with a BMI more than 28 (all patients had BMI more than 30 but had reduced weight before the surgery using a diet), (ii) having flank folding, bulging and excess fat; (iii) having abdominal and flank skin sagging and laxity; and (iv) patient’s request for not extending the incision to the flanks or laterally. The exclusion criteria were (i) high risk of surgery for the patient; (ii) having heart disease, pulmonary and kidney failures; and (iii) being pregnant or having the intention of becoming pregnant in near future. 

**Fig. 2:**
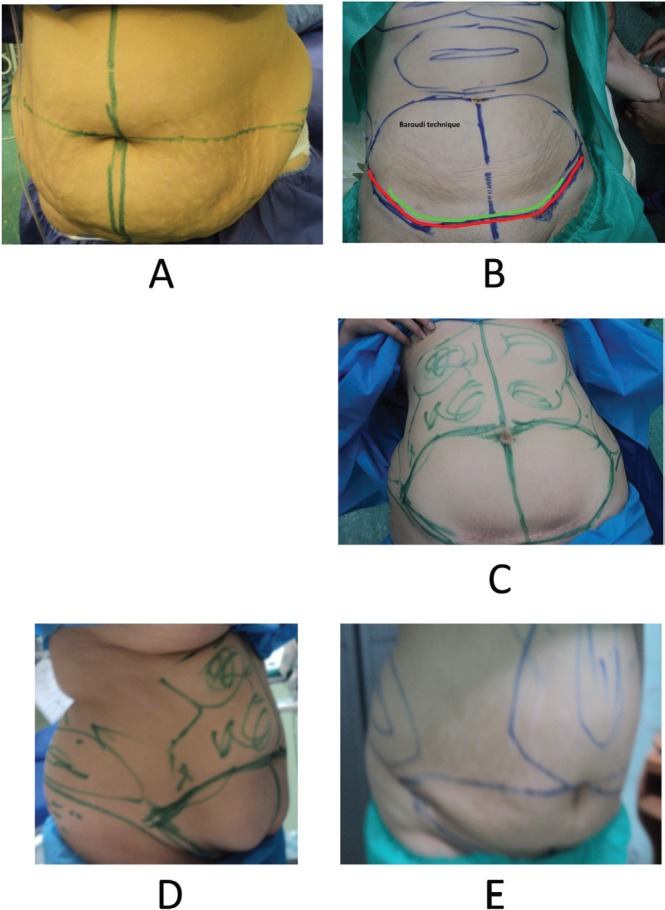
Classic abdominoplasty was designed for the abdominal wall**. **The upper incision line was determined to be one centimeter above the umbilicus based on epigastric skin laxity (excessive laxity and resection). This incision line stretched from the umbilicus to the anterio-superior iliac spine at a mild angle of 15 to 25 degrees.

After selecting the women who met the inclusion criteria, the surgery objectives were explained for them. Cell blood count, platelet, blood urea nitrogen (BUN), creatinine, prothrombin time, partial thromboplastin time, international normalized ratio, and fasting blood sugar (FBS) were determined for all patients. They received advices on cardiac and anesthesia procedures. Classic abdominoplasty was designed for the abdominal wall (Figure 2). It involved designing suction sites in the epigastrium, left and right upper quadrants and right and left flanks. In addition, the surgical incision site was determined using the standard and classic abdominoplasty requirements. The primary incision size was shorter or longer based on abdomen length and flank skin laxity. Previous surgical scars due to caesarian were also included in the resected region. The upper incision line was determined to be one centimeter above the umbilicus based on epigastric skin laxity (excessive laxity and resection). This incision line stretched from the umbilicus to the anterio-superior iliac spine at a mild angle of 15 to 25 degrees (Figure 2). 

**Fig. 3:**
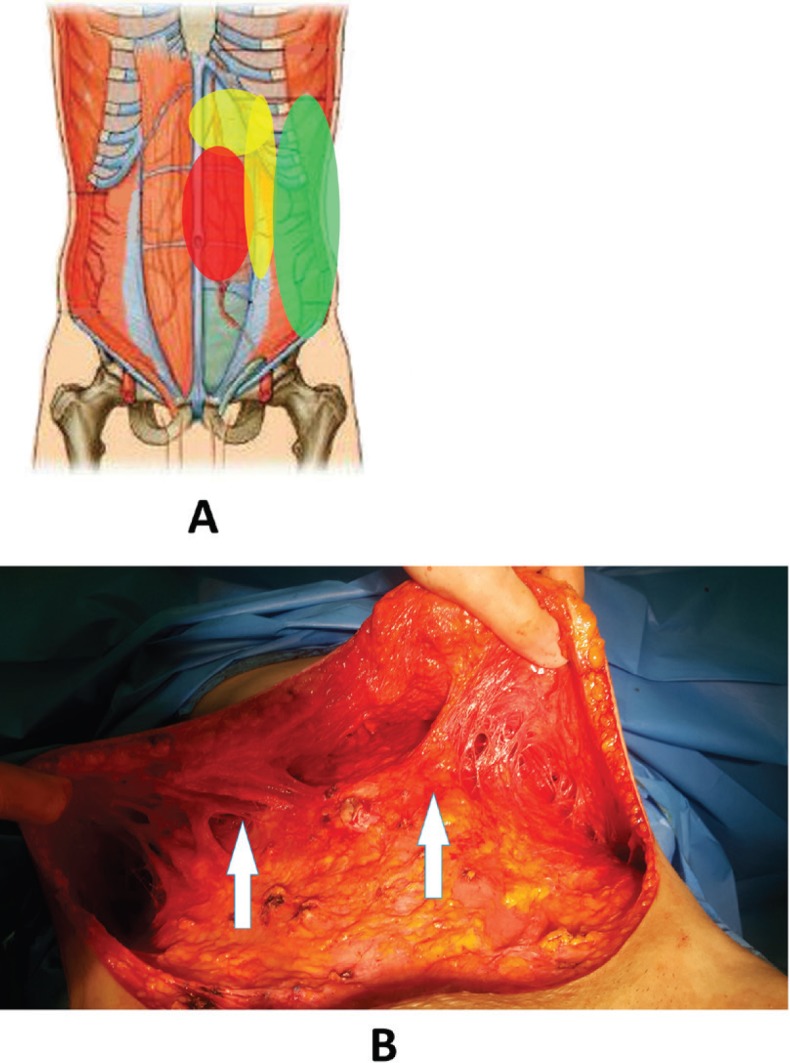
The fat in the epigastric, subcostal and lateral flap regions was removed up to 2 cm pinching test. The paramedian perforator was saved**.**

**Fig. 4:**
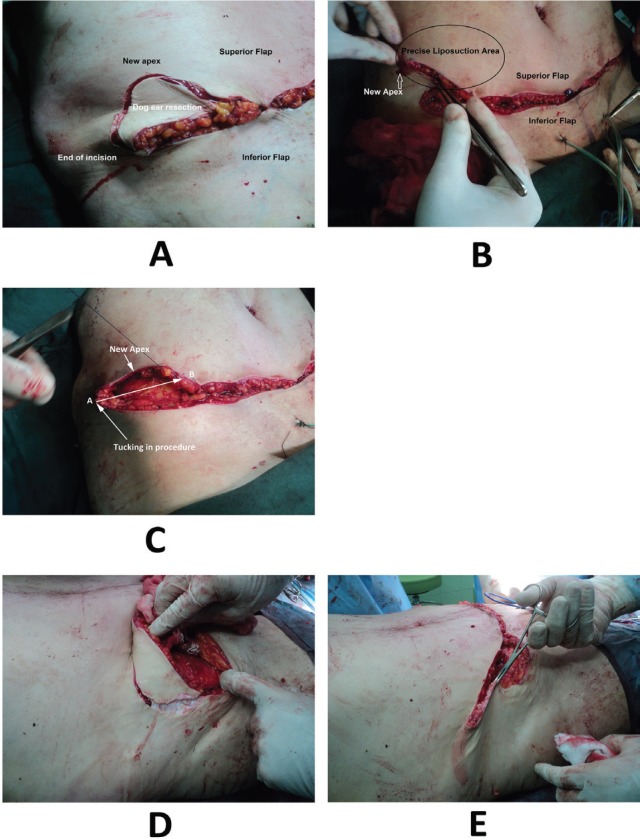
The lateral part of the superior flap was sutured to the medial of the inferior flap to decrease the incision length of superior flap. Up to two third of this flap was tucked in**. **This technique created a new incision apex in the medial border of the iliac crest**. **Thus, the one third and one fourth intersection points on the upper flap were transferred into the inferio-lateral part of the body and the new incision apex was pulled in the medial of iliac crest.

**Fig. 5:**
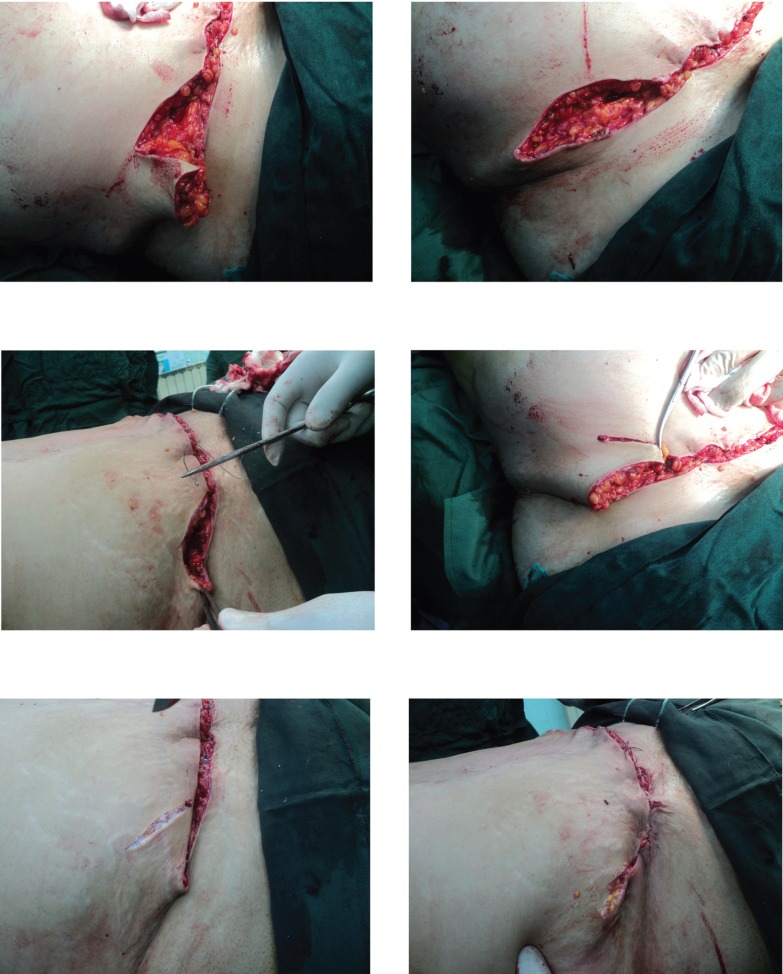
Removing the dog ear in the new technique.

Many obese women had abdominal sagging and laxity which lead to folding in lateral abdomen. On the other hand, wrinkles emerge and intensify abdominal and flank skin folding after extensive fat liposuction in their flank. Hence, to solve this problem several mixed techniques were employed in this study including (i) The fat in the epigastric, subcostal and lateral flap regions was removed up to 2 cm pinching test. The paramedian perforator was saved (Figure 3). However, the flank fat and medial of the iliac crest were completely removed (Figure 2). This technique improved the beauty and contributed to correction of dog ear, skin tags, and wrinkles. All of the skin under umbilicus was resected. The umbilicus was replaced and the superior flap was attached to the inferior flap of the pubis. The lateral part of the superior flap was sutured to the medial of the inferior flap to decrease the incision length of superior flap. Up to two third of this flap was tucked in (Figure 4c). 

(ii) A large part of the dog ear was removed in the one third end of the superior flap. The new incision apex was tucked 5-7 cm into the medial of the inferior flap. This technique created a new incision apex in the medial border of the iliac crest (Figure 4). It corrected the flank laxity and prevented recurrence of dog ear and incision extension into the flank or prevented the tendency of the incision end to form into a panhandle-like shape (Figure 1). The fat in the distal-lower region, medial of the iliac crest and flanks was subjected to a final suction procedure. But the incision appearance looked like a quadric-circle or curvature (Figure 4 and 5). The interesting point about our technique was correction of dog ear, bulging and flank laxity. 

In addition, in the lateral of the inferior flap on the groin, the incision edges were adjusted. Thus, the one third and one fourth intersection points on the upper flap were transferred into the inferio-lateral part of the body and the new incision apex was pulled in the medial of iliac crest (Figure 4). This technique had the following advantages: (i) It prevented formation of a panhandle-shaped incision line and the incision apex did not descend (Figure 4). In Figure 1, it has been shown that incision end has descended inferiorly in surgery by another surgeon. (ii) By repeating the suction in the medial of the iliac crest and flank, removing a large part of the skin excess end and changing the incision apex, the dog ear was corrected properly and an attractive appearance has been shaped in the abdomen (Figure 4). (iii) It removed the flank laxity and improved the appearance of this region. It also gave adequate lumbar curvature. 

In the standard correction, the dog ear is completely removed and the repair procedure starts from the incision apex without tucking in. As a result, the incision increases in length and extends into the flank. On the other hand, after the patient wakes up and stands, folding might emerge. In addition, the fall of the incision apex creates a panhandle-like shape (Figure 5). In addition to the common postsurgical examinations, the patients were fully examined twice and completed a questionnaire. Variables such as age, sex, dog ear, correction of abdominal and flank subcutaneous fat and folding, satisfaction with surgery, folding, and abdominal and flank subcutaneous fat and wrinkles, wearing tight fit clothes, surgical wound infection, hematoma, scar, thromboembolism, and other complications were assessed. 

The scales of scoring dog ear correction after surgery were as follows: (1) weak: skin sagging is more than 2 cm; (2) moderate: skin sagging is between 0.5 and 2 cm; (3) good: little skin sagging or wrinkle less than 0.5 cm; and (4) excellent: no skin sagging or wrinkle. The scales of scoring for the remained skin laxity and bulging after surgery were as follows: (1) weak: the skin wrinkle can be grasped by hand; (2) moderate: there are some skin wrinkles which cannot be grasped by hand; (3) good: there is a mild skin wrinkle which can be overlooked; and (4) excellent: there is no skin wrinkle. The scales of scoring the remained abdomen fat after surgery were (i) weak: more than 4 cm layer of fat is left; (ii) moderate: a 3 to 4 cm layer of fat is left; (iii) good: 1 to 3 cm layer of fat is left; and (iv) excellent: less than 1 cm layer of fat is left. The scales of scoring the remained flank fat after surgery were as follows: (A) weak: more than 3 cm layer of fat is left; (B) moderate: a 2 to 3 cm layer of fat is left; and (C) good: 1 to 2 cm layer of fat is left; 4) excellent: less than 1 cm layer of fat is left.

The data was described and analyzed using descriptive indices and measures of central tendency including frequency, mean, and standard deviation and Chi-Square test. All analyses were done by the statistical package for social sciences (SPSS) software, version 21 (Chicago, IL, USA). 

## RESULTS

A total of 84 women underwent liposuction in our center from May 2014 to February 2016. However, only 25 women who had the inclusion criteria underwent abdominoplasty using our new technique. All women were between 33 and 62 years old (mean age of 47±7.2 years old), while 14 of them (56%) had a BMI of 30 to 33 kg/m^2^ and 11 women (44%) had BMI of 28 to 30 kg/m^2^ at the time of surgery (Table 1 and 2). The average amount of liposuction aspirate was 2,350 mL (1700-3200 mL), and the size of the average excised skin ellipse was 23.62×16.08 cm (from 19×15 to 27×18 cm). Dog ear, skin laxity, bulging and fat deposit correction were assessed and scored in two and four months after the surgery (Table 1). Also, patients’ satisfaction was assessed four months after the surgery (Table 2).

**Table 1:** The frequency of surgeons’ scores for dog ear, skin laxity, bulging and fat deposit correction in two and four months after the surgery.

**Resolved problem**		**Weak** **No. (%)**	**Moderate** **No. (%)**	**Good** **No. (%)**	**Excellent** **No. (%)**
Dog ear correction	2^nd^ month	0 (0)	3 (12)	13 (52)	9 (36)
	4^th^ month	0 (0)	0 (0)	5 (20)	20 (80)
Skin laxity and bulging	2^nd^ month	1 (4)	4 (16)	16 (64)	4 (16)
	4^th^ month	0 (0)	2 (8)	10 (40)	13 (52)
Fat deposit	2^nd^ month	0 (0)	1 (4)	17 (68)	7 (28)
	4^th^ month	0 (0)	0 (0)	12 (48)	13 (52)

**Table 2:** The frequency of patient satisfaction scores of the treatment for dog ear, skin laxity, bulging, fat deposit correction and wearing tight fit cloths in the 4^th^ month after surgery.

**Satisfaction of resolved problem**	**Weak** **No. (%)**	**Moderate** **No. (%)**	**Good** **No. (%)**	**Excellent** **No. (%)**
Dog ear correction	0 (0)	5 (20)	17 (68)	3 (12)
Skin laxity and bulging	0 (0)	6 (24)	16 (64)	3 (12)
Remained Fat deposit	0 (0)	2 (8)	22 (88)	1 (4)
Tight fit cloths	0 (0)	4 (16)	10 (40)	11 (44)

Infection was observed among none of the patients. In two (8%) patients, hematoma was observed in the surgical wound as a result of drain dysfunction. There was partial hematoma which improved through pressure dressing and care (without an explorer or another treatment method). Surgical scars were observed in four (16%) women who healed gradually using an anti-scar ointment (Rejuderm, Iran) until six months. Therefore, there was no need to correct the surgical scars in any participant. In two women, there was a need for blood injection due to the postoperative blood pressure drop which was probably caused by intraoperative hemorrhage and massive liposuction (Table 3). 

**Table 3:** The frequency of encountered adverse effects after the surgery.

**Post-operation adverse effects**	**No.**	**%**
Wound infection	0	0
Hematoma	2	8
Scar	4	16
Blood injection	2	8
Peptic ulcer	1	4

Twenty-four (96%) women were discharged within one day after surgery and only one woman stayed for two days because of blood pressure drop and the need for blood transfusion. A woman (4%) experienced abdominal pain on the 2^nd^ day after the surgery. She had symptoms of peritoneal irritation. In the upright and horizontal radiographs, the patient’s abdomen showed entrapment of air under the diaphragm. Hence, the surgery was repeated. After opening the patient’s abdomen from the place of the previous incision, it was found that the region was full of fibrin. The abdominal exploration revealed existence of perforated peptic ulcer. Hence, the patient’s abdomen was completely washed and the peptic ulcer was sampled and repaired using a 0-2 silk thread and omental patches. Further investigations showed that the problem was caused by self-curing with non-steroidal anti-inflammatory drugs (diclofenac rectal suppository two times per day) from one day after surgery as well as surgery stress. 

Seroma was observed in none of the patients. However, it was not removed until the secretions amount decreased to less than 30 mL because of drain placement. Excessive and irritating secretions were also seen in any patient. There was no sign of fat embolism or thromboembolism, but one woman had symptoms of dyspnea. After examinations and consulting sessions, it was found that there was no pulmonary embolism and she was released one day after the surgery. In spite of extensive suction, no flap necrosis was reported. The important finding about the beauty aspect of the surgeries was that the women were satisfied with wearing tight fit clothes. Our results showed that 4 (16%) and 21 (84%) women were moderately and completely satisfied with the surgeries.

**Fig. 6:**
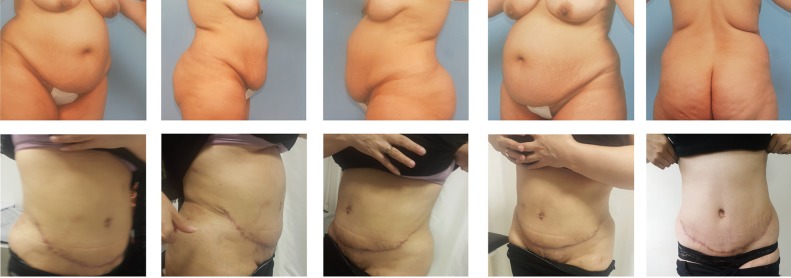
Comparing the result of using this new technique, four months after the surgery with the conditions before surgery.

The follow-up two months after surgery showed that there was a difference between frequencies of dog ear correction in patients with BMI more and less than 30 kg/m^2^ which was not significant (*p*>0.05). No significant relationship was observed four months after the surgery in this regard too (*p*>0.0). There was a significant difference between BMI and the remained abdominal and flank fat deposit two months after the surgery (*p*=0.05). However, no significant difference was observed between removing flank and abdominal fat and BMI four months after the surgery (*p*=0.06, Figure 6).

## DISCUSSION

The goal of abdominoplasty is to correct the abdomen anatomy and create the beauty demanded by the patient. Our technique can correct the dog ear and complete flank and abdominal fat suction. Moreover, abdominal and flank folding are corrected considerably and adequate lumbar curvature is observed. All of these achievements are the result of removing the skin excess end and changing the incision apex (Figure 4), making changes in the standard abdominoplasty technique, and precise liposuction. At the end of the 4^th^ month, 86% of the patients were completely satisfied with the surgery and 14% were moderately satisfied.‌ In spite of mass aggressive liposuction and dog ear resection, we had no flap ischemia or necrosis. 

In different studies, abdominoplasty complications such as folding, remaining lateral and abdominal fat, and dog ear were still problems especially in obese patients. The patients have sometimes undergone other surgeries to improve these conditions.^2^^,^^3^^,^^7^ Evidently, repeated surgeries on a patient who undergoes cosmetic surgery can result in unsatisfactory outcomes that can negate the efforts made by the surgery team. 

Neaman and Hansen found an overall complication rate of 37.4% in their study on abdominoplasty. Major complications, including hematoma requiring surgical intervention, seroma requiring aspiration or surgical drainage, cellulitis or abscess requiring hospital stay and intravenous antibiotics, deep vein thrombosis, and pulmonary embolism, occurred in 16% of their patients. The rate of minor complications (hematoma or seroma requiring no intervention, epidermolysis, small-wound dehiscence, neuropathic pain, and minor cellulitis) was 26.7%. So obese patients had a significantly increased risk of developing major complications compared to non-obese patients (53.4% versus 28.8%, *p*=0.001).^8^ In our study which was on obese patients, the rate of complication was 8%, although we did aggressive liposuction. However, this led to hematoma. 

In a retrospective research by Mataraso and colleagues on 400 patients, 24 patients were subjected to repeated abdominoplasty. Moreover, seven patients (29%) were re-operated due to development of dog ears and one patient (4%) was operated again due to a complication. They concluded that abdominoplasty is a completely approved surgical procedure. They stated that the public demand for abdominoplasty is increasing for two reasons: (1) increased population age, and (2) low complications and risks of this surgery. Hence, improving this surgery to reduce delayed complications such as dog ear in patients is substantially important.^2^ Although it is possible to repeat the surgery for a patient, it is important to employ the right surgical method to avoid repeated surgeries. Their study did not propose a new surgical technique for reducing side effects and consequences. However, our research has explored a new abdominoplasty technique knowing that side effects can cause such problems. In our study, there was no major complication in need of repeated operations or delayed surgery for correction of the dog ear, seroma or liposuction. 

In the past decades, precise liposuction has evolved and become more popular.^9^^,^^10^ In a research by Hafezi and colleagues, 56 patients were exposed to extensive lumbar and abdominal liposuction operations. A main goal of their research was to prevent spread of the incision to the back and to limit the incision to anterio-superior iliac spine on the flanks. Their results showed a decrease or elimination of dog ear and lumbar curvature formation. There was also no sign of necrosis and dehiscence of the edges of incision or a sign of thromboembolism and fat embolism in spite of precise liposuction. Their results and ours indicated that extensive flank and abdominal liposuction is risk-free.^5^ However, the incision and flap dissection methods differed in these two studies. They did limited dissection on the upper area of abdomen, but in our study, the vascular pedicle was preserved to allow for better blood flow. At the end of the dissection, the flap end of skin excess was removed in our study. This action drastically eliminated excess folding and dog ears. Results of Hafezi and colleagues and ours are similar in terms of a decrease in dog ear formation and flank curvature.

In 2006, Stewart *et al.* investigated the side effects of abdominoplasty in 278 patients who were operated by four surgeons within five years. In their study, 18% of the patients reported early complications while 25% experienced delayed side effects. Of which, 12%, experienced dog ear, 10% subcutaneous fat asymmetry and 8% irritating scars. It is clear that the resulting complications especially the dog ear and folding and repeated corrective abdominoplasty are unpleasant and sometimes unacceptable for patients.^11^ In another study, mixed abdominoplasty was used to correct the dog ear and flank skin laxity. In this research, which was conducted for five years using the modified W technique, it was found that the aforementioned technique reduced these two complications.^3^

The drawing of a standard grid on the abdomen before the surgery was previously studied. The results showed that this technique created surgical scar symmetry and precisely determines skin resection before the operation. However, different abdominoplasty techniques are modified during surgeries by surgeons.^1^ The grid design gives the surgeon the required insight and exceptional ability to select the incision technique. It simplifies the surgical scar and reduces delayed side effects of abdominoplasty such as the dog ear. Results of our study showed that four months after the surgery, there was no sign of dog ear. Moreover, all of our patients were satisfied of lateral and abdominal fat correction (48% satisfactory and 52% excellent). 

In the investigation by Omranipour and colleagues, the average duration of hospital stay was 3±0.8 days, but in our study, this was approximately one day. In addition, in their study 1% of patients needed blood injection (transfusion), 2% experienced surgical wound infection, 3% displayed flap ischemia, and 20% demonstrated surgical wound roughness.^7^ However, in our study, there was no report of surgical wound infection, dehiscence and flap ischemia although complete and precise liposuction was done because we preserved preumblical perforator vessels or paramedian vessels. Transfusion and limited hematoma were observed in 8% of patients because of extensive lateral and lumbar liposuction. The result was a more beautiful abdomen shape. However, the chances of outbreak of hematoma and the need for transfusion could have increased.

Patronella has insisted on liposuction of adjacent lateral (non-abdominal) areas to ensure harmonious proportion. This series of refinements adds precision and detail by redefining the native anatomical nuances of the abdomen, an aesthetic objective that has been consistently achieved in BMI between 20 and 35.^6^ Also, Nunes da Costa and Matias reported that if you do suction or deep fat excision, it does not lead to flap necrosis in abdominoplasty.^12^ Because of aggressive liposuction and dog ear resection, another technique to reduce local complication is using progressive tension suture that has been reported by Pollack and Pollack.^13^ Our average amount of liposuction aspirate was 2,350 mL. This was more than the studies of Kim and colleagues and Weiler and colleagues which were 1,400 mL and 2166.09 mL, respectively.^14^^,^^15^

Seth *et al.* found 23% complications in their study which primarily consisted of wound- and scar-related complications (15.3%) in patients with a BMI of 27 kg/m^2^ who had underwent abdominoplasty.^16^ In a meta-analysis, Winocour and colleagues studied 25,478 abdominoplasty cases in a database. 4% of them (1,012 cases) had encountered complications. Of these, 31.5% were hematomas, 27.2% infections and 20.2% suspected or confirmed venous thromboembolism. The significant risk factors included male sex (relative risk, 1.8), being more than 55 years old (1.4), BMI more than 30 kg/m^2^ (1.3), multiple procedures (1.5), and doing the procedure in a hospital or surgical center versus office-based surgical suite (1.6). Combined procedures increased the risk of complication.^17^

Bertheuil *et al.* described a new technique in 25 abdominal body-contouring reconstructions following massive weight loss. They were treated by circumferential lipo-body lift. The mean pre-body lift BMI was 26.71 kg/m^2^; minor complications including wound dehiscence, wound infection, and fat necrosis that were reported in 40% of patients. They concluded that this novel technique was less invasive than the traditional lower body-lifting method.^18 ^Understanding the mechanism of dog ear formation is necessary to facilitate treatment. There are many solutions, but in all cases, the correction involves extending the scar and the sacrifice of the dermal plexus. In our study, we did not extend the scar or incision. Jaber and colleagues described a novel technique of dog ear correction by using a three-bite suture that sequentially pierces the deep fascial plane and each dog ear’s margin, thus allowing for flattening the dog ear by anchoring the over-projecting tissue to the deep plane.^19^

Also, Kishi *et al.* described a new technique for correcting dog ear that allows correction sufficiently in the primary surgery. Its principle is to de-epithelialize the dog ear portion and dig it into the dermis to preserve the subdermal plexus at the base of the flap intact. They stated that this method was useful especially at the base of a transposition flap because the pedicle tended to be narrowed by the conventional correction.^20^ But in our technique, we resected the dog ear and the final shape of the dog ear was L formed (semi-circular). In our study, we paid special attention to doing suction in areas where it had cosmetic results (Figure 3 and 5). Our overall good and excellent satisfaction with results was 83% (Table 2). Our findings revealed improvement in abdominoplasty results and a decrease in complications such as folding and dog ear. Other results were prevention of spread of the incision to the lateral and back parts and formation of proper lumbar curvature. 

If we use a long incision that extends laterally and superiorly at first, we are obliged to tuck in the incision apex more than the routine into the medial of the inferior flap for dog ear and flank skin laxity correction. This extends the new apex end superiorly. Thus, the final shape of the incision becomes semi-circular while we want it to become quadrant-circle. This semi-circular incision shape is unsatisfying for the patients. To prevent extension of the incision apex to the upper area more than the line that goes from iliac crest to umbilicus, it is recommended that the end of the lower incision to be 3 to 4 cm before anterio-superior iliac spine in the pilot plan (the length of the primary incision should be smaller than the final incision). This prevents upward extension of the incision at the end of the operation after correcting the dog ear and flank skin laxity. We did aggressive liposuction in the flank, abdomen and subcostal and checked the remained fat with pinching during the operation. We saved the perforator vessels. We had no flap ischemia or necrosis. 

We can conclude that aggressive abdominal and flank liposuction can be safely done when paramedian perforator is preserved. This has a good cosmetic result in the abdomen and flank and prevents bulging in the incision end and flank. The other primary outcome was that to resolve dog ear and flank skin laxity, we must choose the first incision short and then after transferring the superior flap to the inferior flap, we can resect the dog ear. Then the incision size will not extend superiorly or laterally. Using this abdominoplasty technique is recommended on patients with high BMI. 
